# When Is “Type I” Ovarian Cancer Not “Type I”? Indications of an Out-Dated Dichotomy

**DOI:** 10.3389/fonc.2018.00654

**Published:** 2018-12-21

**Authors:** Carolina Salazar, Ian G. Campbell, Kylie L. Gorringe

**Affiliations:** ^1^Peter MacCallum Cancer Centre, Melbourne, VIC, Australia; ^2^Sir Peter MacCallum Department of Oncology, The University of Melbourne, Parkville, VIC, Australia

**Keywords:** epithelial ovarian cancer, type I-II tumors, dualistic model, classification, ovarian carcinoma

## Abstract

The dualistic classification of epithelial ovarian cancer (EOC) into “type I” and “type II” is widely applied in the research setting; it is used as a convenient way of conceptualizing different mechanisms of tumorigenesis. However, this classification conflicts with recent molecular insights of the etiology of EOC. Molecular and cell of origin studies indicate that while type II tumors could be classed together, type I tumors are not homogenous, even within the histological types, and can have poor clinical outcomes. Type II high grade serous carcinoma and type I low grade serous carcinomas best fit the description of the dualistic model, with different precursors, and distinct molecular profiles. However, endometriosis-associated cancers should be considered a separate group, without assuming an indolent course or type I genetic profiles. Furthermore, the very clear differences between mucinous ovarian carcinomas and other type I tumors, including an uncertain origin, and heterogeneous mutational spectrum and clinical behavior, indicate a non-type I classification for this entity. The impression that only type II carcinomas are aggressive, have poor prognosis, and carry *TP53* mutations is an unhelpful misinterpretation of the dualistic classification. In this review, we revisit the history of EOC classification, and discuss the misunderstanding of the dualistic model by comparing the clinical and molecular heterogeneity of EOC types. We also emphasize that all EOC research, both basic and clinical, should consider the subtypes as different diseases beyond the type I/type II model, and base novel therapies on the molecular characteristics of each tumor.

## Introduction

Epithelial Ovarian cancer (EOC) is one of the four malignancies of the female genital tract (1) and ranks fifth in deaths caused by cancer among women (2–4). The median age of EOC patients is 63 years (5) and persistent pelvic, abdominal and back pain, unusual bloating, frequent urination and lack of energy are all EOC symptoms related to everyday conditions for women in this age group (6, 7). The non-specific symptoms of EOC is one of the explanations for generally advanced-stage disease at diagnosis (8) which is associated with less favorable prognosis, abdominal metastasis and recurrence within 18 months for most patients with advanced disease (5).

EOC comprise diverse types of histology, and over the years a number of classification systems have been proposed. One system that is widely applied in the research setting is a dualistic classification into “type I” and “type II” cancers (9). However, this system was devised prior to the availability of very large data sets of base-pair level genomic information for EOC, and seems incompatible with the latest molecular insights of the origin and relationships of the various forms of EOC. In this review, we argue that this dualistic model is widely misinterpreted when applied across the full spectrum of EOC and fails to incorporate the clinical and molecular heterogeneity of this disease. Application of this framework to study design and interpretation has led to many studies in contexts as varied as transgenic mouse models (10), biomarker studies (11), clinical research (11, 12), and biological analysis (13). By using the dualistic model, these studies have likely missed true biological signals by merging at least four distinct subtypes into a single Type I grouping.

### History of EOC Classification

**Figure 1:**
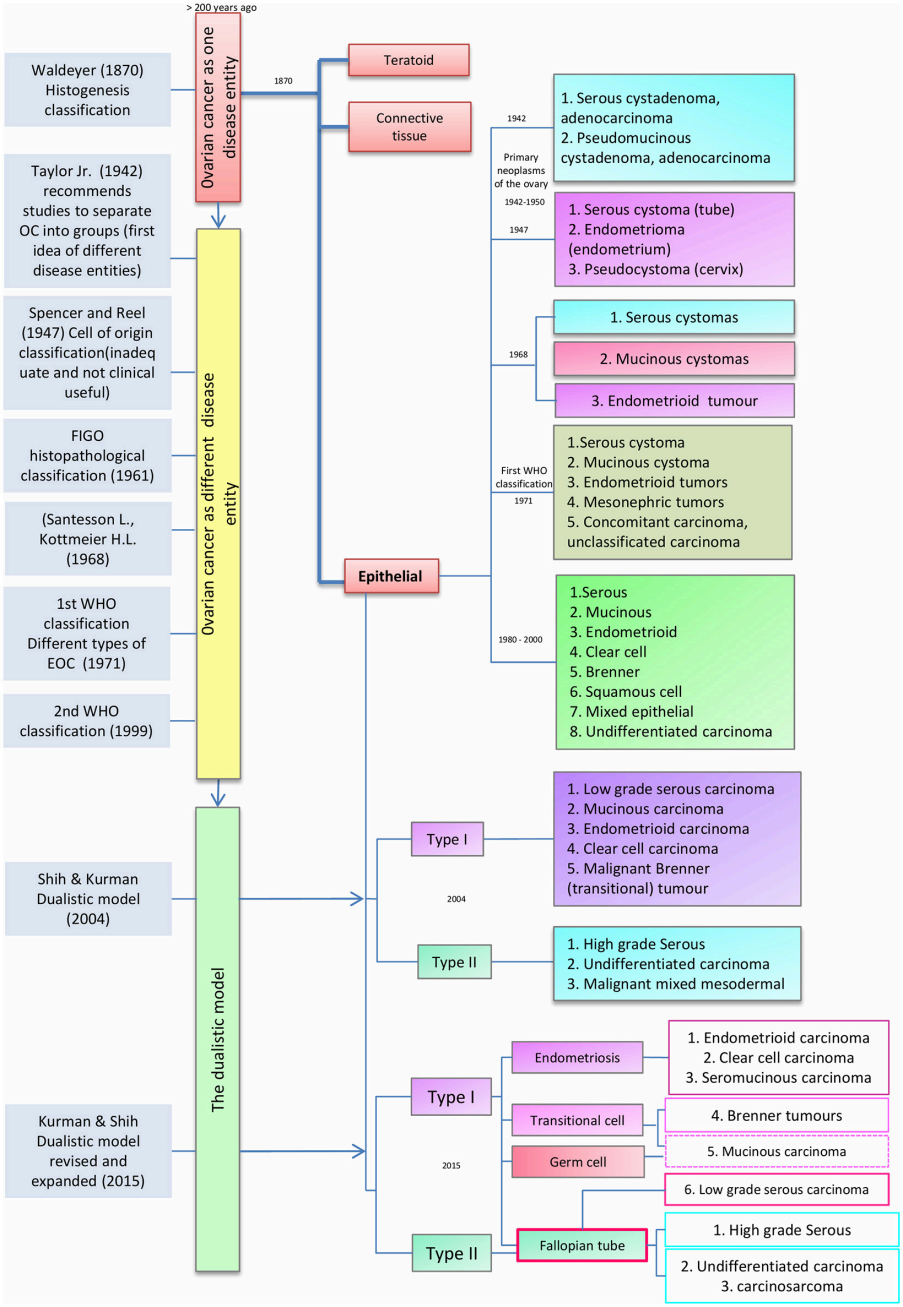
Timeline classification of epithelial ovarian cancer. Blue boxes on the left show author contributions to ovarian cancer (OC) classification over time. Red, yellow, and green boxes represent the evolution of OC classification from a single disease entity to a binary model. Finally, the different sub classifications of epithelial ovarian cancer (center red box), are presented from 1942 to the latest model in 2015.

As noted by Peaslee in 1873, for more than 200 years ovarian cancer (OC) was classified according to tumor size and character into cystic and solid tumors (14, 15). In the late nineteenth century, improvements in pathological techniques led von Waldeyer-Hartz (16) to the first classification based on histogenesis of ovarian tumors; differentiating epithelial and teratoid tumors from connective tissue growths. Subsequently, others have proposed several histogenesis-based classifications (14) (Figure 1).

In 1942, Taylor Jr. noted that research studies and clinicopathological reports had to take account of the specific type of OC. This idea was supported by Marchetti, who in 1950 recommended a standardized system to classify OC whereby epithelial tumors were part of the “primary neoplasm of the ovary” and divided into two groups: (1) serous cystadenoma and cystadenocarcinoma and (2) Pseudomucinous cystadenoma and cystadenocarcinoma. Subsequently, in 1947, Spencer and Reel proposed a modification of the classification described by Schiller (1940), in which EOC was classified on the origin of tumors into three groups: (1) serous cystomas (tubal origin), (2) endometrioma (endometrial origin) and (3) pseudocystoma (cervical origin) (14, 17). This classification system was ahead of its time given the limited data that was available about the cellular origins of OC. In 1961 this model was supplanted by an ovarian-centric histopathological classification as proposed by the Cancer Committee of the International Federation of Gynecology and Obstetrics (FIGO). The FIGO model mandated that all cases of neoplasms producing hormones, germ cell tumors and metastatic carcinomas from non-ovarian primaries should be excluded from therapeutic statistics on ovarian epithelial tumors (18). In 1968, Santesson and Kottmeir proposed EOC as a group of diseases instead of one entity and stated that treatments should be based on homogeneous groups of tumors. From that time, EOC primary classification consisted of serous, mucinous and endometrioid tumors with each type subclassified into benign, borderline, or malignant (19).

The first widely used clinical histological classification of the common primary epithelial tumors of the ovary was developed by the World Health Organization (WHO) in 1971 and divided EOCs into serous cystomas, mucinous cystomas, endometrioid tumors, mesonephric tumors, concomitant carcinoma, and unclassified carcinoma (18). This classification was endorsed without modifications until 1999 when the WHO published a new classification in which EOCs were grouped into serous, mucinous, endometrioid, clear cell, Brenner, squamous cell, mixed epithelial tumors and undifferentiated carcinomas (20). These subtypes are defined according to the histological appearance of the epithelium; i.e., serous carcinomas resemble the serous epithelium of the fallopian tube, mucinous carcinomas have mucin-containing cells, clear cell tumors have cells with a clear cytoplasm and “hobnail” cells, endometrioid carcinomas have epithelium similar to the glands of the endometrium, and Brenner tumors have transitional cell aggregates. Undifferentiated carcinomas generally lack these defining cell types and are extensively atypical and highly proliferative (21, 22). However, the absence of reliable knowledge about the origin and model of progression of EOC meant that the etiology of EOC was not taken into account in the revised WHO classification.

### The Dualistic Model

Since 1942, the idea that OC does not represent a single disease and should be classified according to clinicopathologic types and groups became increasingly clear. Fukunaga et al. suggested that EOC development pathways are different for each subtype. For example, endometrioid ovarian carcinomas were shown to have an origin in endometriosis, instead of the ovarian surface epithelium (23). This concept was supported by Obata et al. who demonstrated that *PTEN* alterations are common in endometrioid carcinomas, contrary to serous or mucinous carcinomas (24). Shih and Kurman (9) proposed a dualistic model, using molecular and morphological features rather than predominantly histological appearance. They noted that EOC origin and pathogenesis was poorly understood and specifically its classification was inconsistent regarding the relationship between borderline tumors and invasive carcinomas. Under the dualistic model, the third WHO EOC classification (2000-2005) instead grouped EOC into two broad categories: type I and type II tumors, corresponding to tumorigenic pathways and not to specific histopathology. Type I tumors included low grade serous (LGSC), mucinous (MOC), endometrioid (ENOC), clear cell carcinomas (CCOC), and malignant Brenner (transitional) tumors, while type II included high grade serous (HGSC), undifferentiated carcinomas and malignant mixed mesodermal tumors. Type I tumors appeared to have clearly defined precursor lesions, cystadenomas, atypical proliferative tumors, and non-invasive carcinoma, and evolved along a pathway that resembles the adenoma-carcinoma sequence described for colorectal cancer. The evolution of type I tumors was described as slow and associated with molecular changes in *BRAF, KRAS*, and *PTEN* that were not found in type II. In contrast, type II tumors were defined as rapidly evolving from the ovarian surface epithelium or inclusion cysts but lacking morphological precursors, carrying *TP53* mutations and with early metastatic spread (9, 25).

The dualistic model is not used clinically in real-world settings, whereas WHO and FIGO classifications are important criteria for interpretation of clinical data and deciding EOC therapy (21, 22, 26). In 2014, the WHO updated the classification of tumors of the female reproductive organs based on the newly understood biology of ovarian tumors. The idea that OC is a highly heterogeneous disease in terms of etiology, histological, epidemiological, clinical and molecular features is no longer controversial and this heterogeneity suggested a classification according to the different epithelial cell types present in the reproductive female tract. In the previous WHO classification, the origin of all EOC was considered to be the mesothelial surface of the ovary but in the new classification tubal carcinogenesis is cited as the point of origin for high-grade serous carcinomas. Although the origin of mucinous and some advanced serous carcinomas is still unclear, the new classification is more consistent with our current understanding of the disease. The new classification removes transitional cell tumors (21, 22) but includes seromucinous tumors as a new type [Kurman et al. (27) designated them as mixed Müllerian tumors]. The intermediate step from benign to invasive lesions remains as borderline tumors.

**Figure 2:**
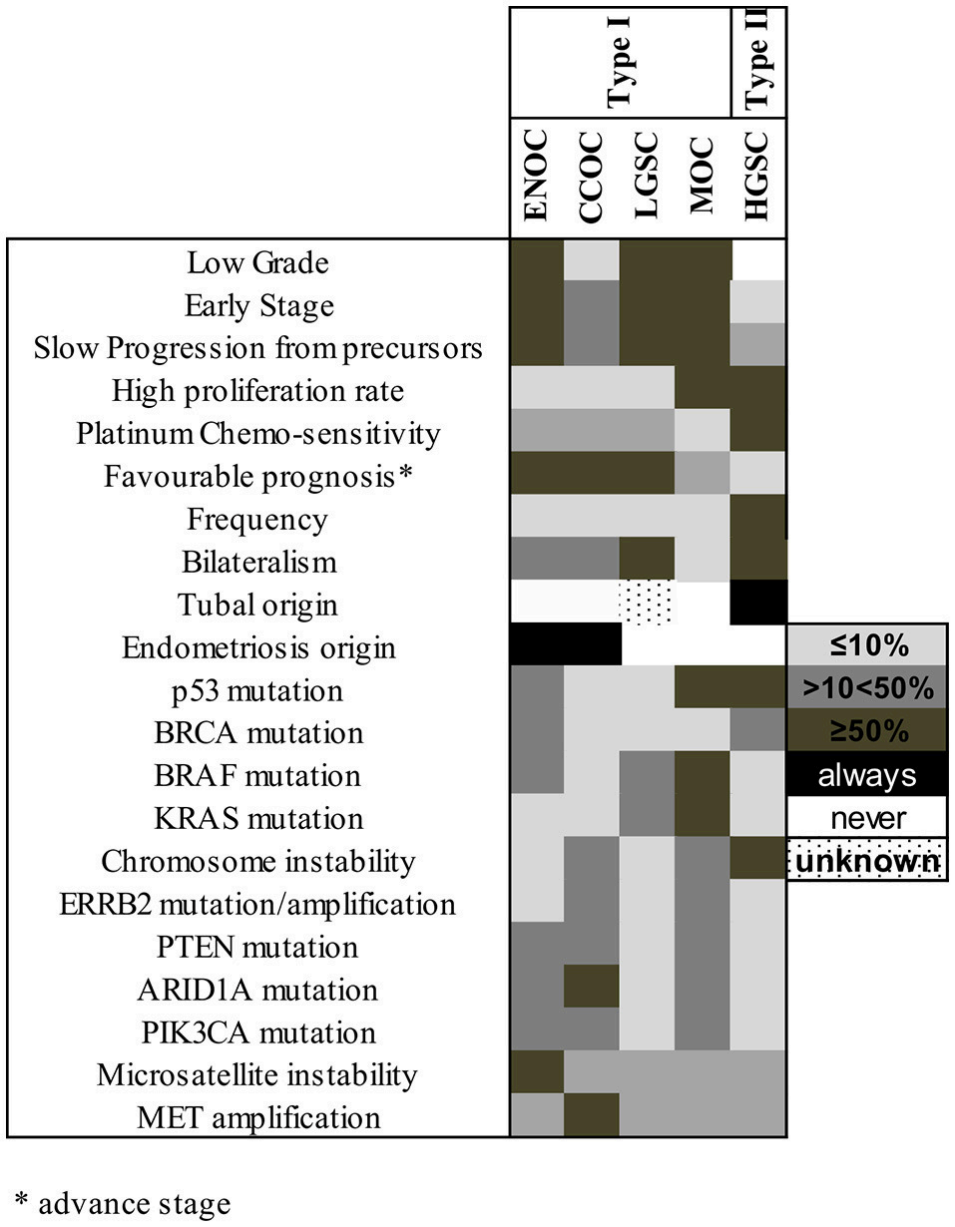
Molecular factors and characteristics distinguishing type I and II tumors. The shade of each box represents the frequency of the characteristics listed at left in each subtype, grouped according to their previous type I/type II assignment. ENOC, endometrioid ovarian carcinoma; CCOC, clear cell ovarian carcinoma; LGSC, low grade serous carcinoma; MOC, mucinous ovarian carcinoma; HGSC, high grade serous carcinoma.

Recent genetic and histopathological studies, as well as the new WHO classification for EOC, prompted an update of Kurman and Shih's dualistic model. Type I tumors now include MOC, LGSC, seromucinous, ENOC, CCOC, and Brenner (transitional) tumors (Figure 1), while type II tumors include HGSC, carcinosarcomas, and undifferentiated carcinomas (27). Genetic stability and mutational profiles are key molecular factors distinguishing type I and II tumors: type II have high chromosome instability and carry *TP53* mutations. Somatic mutations in *PIK3CA, PTEN, CTNNB1, KRAS, BRAF*, and *ARID1A* are frequent in type I tumors, whereas chromosomal instability affecting genes like *CCNE1, RB1*, and *NF1* are common in type II (27) (Figure 2).

The dualistic model is conceptually attractive and a convenient way of describing different mechanisms of disease initiation and progression. However, this model has led to the inaccurate impression that only HGSC is aggressive, has poor prognosis and carries *TP53* mutations (5, 25, 28) while type I tumors are relatively homogenous and less aggressive. It is now clear that, particularly for non-HGSC, these groups are far from homogenous, even within their histological types, and many can be clinically very aggressive as described below.

### Type I Tumors and MOC

Type I tumors are currently thought to arise from benign precursor lesions of the ovarian surface epithelium (29, 30) or the fallopian tube (31, 32) (LGSC), or endometriotic implants in the pelvis (CCOC, ENOC) (33–35). The exact cell of origin of LGSC is still unclear and more studies are needed to determine whether the ovary or fallopian tube or potentially both are the source. Clear cell and endometrioid carcinomas share similar mutational profiles however; the histological differences suggest that the cells of origin could be different. Cochrane et al. showed that clear cell carcinoma and endometrioid carcinoma arises from different types of cells, ciliated and secretory cells, respectively, (36). Seromucinous tumors are also thought to be derived from endometriosis at a similar frequency as endometrioid and clear cell tumors (37). Type I tumors are best exemplified by LGSC, which indeed fit many of the characteristics, with clearly defined benign and borderline precursor tumors, mutations in *KRAS* or *BRAF* but not *TP53*, stable chromosomal profiles, and low proliferation rates (38). However, features of the other type I tumors vary widely.

Comprehensive genome-wide analyses have shown that inactivating mutations in the *ARID1A* gene occur at high frequency in ENOC and CCOC (30 and 57%, respectively) (39). *TP53* mutations are less frequent, but not absent, in CCOC (~5–10% in recent studies) (40–43) while *PIK3CA* mutations are common. ENOC have a similar pattern of genetic aberrations to CCOC, with prevalent *ARID1A, PIK3CA*, and *CTNNB1* mutations and a low rate of *TP53* mutations (44, 45). A recent whole genome analysis identified *TP53* mutations in 28% (8/29) of ENOC (43). Interestingly, ENOC and CCOC can present with microsatellite instability, rarely detected in other ovarian cancers (46, 47). Copy number data from ENOC are limited, with a substantial percentage of high-grade ENOC cases reported in older studies likely to be misdiagnosed HGSC (48). CCOC have more copy number events than serous borderline tumors (49) including frequent gain of 8q (50), although less than HGSC. Some studies have shown that a subset of CCOC has a high level of chromosome instability, and this profile correlates with a worse prognosis (51, 52). Therefore, the endometriosis-related ovarian carcinomas may themselves be heterogeneous and subsets may carry type II characteristics.

MOC is the clear outlier in the type I group: its origin remains unclear and it behaves differently to the other histologic subtypes of EOC. Epidemiologic studies have suggested ovulation as a factor for developing EOC (53), whereby oral contraceptives reduce the risk of EOC (54) and menarchal age, nulliparity (55), estrogen exposure, lack of breastfeeding and tubal sterilization are all associated with ovarian cancer risk. In contrast, these factors are only weakly, if at all, associated with MOC, whereas smoking is a risk factor (25, 56–60). Primary MOC commonly express CK7, CEA, CDX2, and CA 19.9, however CK20 positivity is sometimes observed, unlike other type I tumors (56). Most MOC do not express ER or PR, consistent with a non-Müllerian origin (27), but positivity is seen in a subset (56, 61), along with the Mullerian marker PAX8 in 50% of MOCs (62, 63). MOC tumors predominantly have gastrointestinal-type differentiation but the endo-cervical type is sometimes seen (64). They can also present with a range of histopathological grades, from mostly borderline with small foci of invasion, to tumors with large areas of high architectural complexity through to a loss of differentiation, infiltrative growth and extensive nuclear atypia (grade 3). In keeping with a type I origin, *KRAS*-activating mutations are present in ~65% of MOC, representing the most common molecular genetic alteration (65–67). Additionally, RAS/MEK pathway activation is frequent in this cancer, since >90% of MOCs have *ERRB2* amplification and/or *KRAS* or *BRAF* mutations (68). Notably different from other type I tumors, 50% of MOC carry a *TP53* mutation (27, 68) which is in stark disagreement with a type I model for MOC, and it is unclear why these events have not been commonly reported in the previous literature. Other genetic events, such as *RNF43, PIK3CA*, or *ARID1A* mutation, are present in < 20% of cases (66, 68).

Inter-tumoral heterogeneity is almost a hallmark of MOC and is reflected in the multiple hypotheses for its origin. One suggestion is that these tumors are only ever metastases derived from occult extra-ovarian carcinomas, however, the existence of mucinous benign and borderline precursors as well as early stage tumors with excellent outcomes suggests this is cannot commonly be the case. MOC has been shown to share key genetic events with benign and borderline tumors (66, 68) but the cell of origin of these is not known, with suggested origins including the ovarian surface epithelium, metaplastic transformation of ovarian inclusion cyst epithelia (69), paraovarian epithelial nests of the tuboperitoneal junction as well as development from Brenner tumors or teratomas (25, 34). Mucinous cystoadenomas have been shown to be potential precursor lesions for MOC; studies revealed identical *KRAS* mutation at codons 12 and 13 in cystadenoma, atypical proliferative and mucinous carcinoma (65, 70). MOC and Brenner tumors share a 12q14-21 amplification and a possible tuboperitoneal junction origin (71); this could be the reason why small MOC tumors can be misdiagnosed as Brenner tumors. However, Brenner tumors could also derive from cystadenomas (27). Recent studies showed that the mucinous epithelial expansion in Brenner tumors leads to the development of cystadenomas and there is a clonal relationship between the mucinous and Brenner components (72, 73). Atypical Brenner tumors also have frequent deletion of *CDKN2A*, a genetic feature characteristic of MOC (74). Similarly, teratomas have also been shown to share a clonal origin with co-existing MOC (72, 75). Thus, genetic and other evidence suggests multiple potential origins for MOC: perhaps the heterogeneity of the disease is reflective of different sources, some of which may remain to be identified.

### Type II Tumors

Recent studies where the entire fallopian tube and fimbria were closely examined have provided strong evidence that HGSC develops from an intraepithelial carcinoma in the fallopian tube fimbria (STIC). Sixty percent of women diagnosed with sporadic HGSC also had STIC (27, 76–78). Moreover, this tubal origin was supported by epidemiological studies were women with intact fallopian tubes have a higher risk of developing HGSC compared to women who had a previous salpingectomy (79, 80). Further evidence has been obtained from genome-wide mutation and gene expression data, clearly supporting a tubal origin for HGSC (81–83). A small subset of HGSC may develop from borderline or LGSC precursors (31, 84–86), but this is rare. The origin of the other variant of type II tumors, solid pseudoendometrioid transition tumors (SET), is still unclear but may develop from either STIC or another tubal precursor (87, 88).

HGSC tumors are frequency diagnosed at an advanced stage, and almost 100% of cases show a *TP53* gene mutation (89, 90). Recent studies have shown that somatic and germline *BRCA* mutations are common in HGSC (83, 88) and The Cancer Genome Atlas study has shown that *CCNE1* amplification, and aberrant NOTCH3 and FOXM signaling are also frequent (91). SET type tumors, which are reported in younger women, have a higher mitotic index and number of tumor-infiltrating lymphocytes than HGSC. Moreover, SET tumors have a yet higher *BRCA1* mutation rate (~17%) and have a better outcome due to greater chemosensitivity (27, 88, 92, 93). Undifferentiated ovarian carcinomas are uncommon and thought to be poorly differentiated HGSC or high grade ENOC although no substantive molecular genetic studies have been reported on this type (34). Undifferentiated carcinomas share the same molecular profile as HGSOC including overexpression of p53, indicating that most probably correspond to HGSC (94–96). Carcinosarcomas (Malignant Mixed Müllerian Tumor) which are biphasic tumors with a carcinoma and a sarcoma component, show frequent *CDKN2A* overexpression and *TP53* mutations (>90%)(9).

### The Dualistic Model Is Not Informative

Independent retrospective studies that have evaluated the prognostic and clinical value of the dualistic model showed that this classification does not correlate with patients' prognosis (94–97). Panici et al. demonstrated that type II EOC present more frequently at an advanced stage than type I tumors and require more aggressive surgery (96). However, multiple studies have shown that the survival rate of patients diagnosed with type I and type II tumors is not significantly different when factors like stage and age are taken into account (96). For example, while over 80% of MOCs are diagnosed at early stage and have a better prognosis than HGSC, when detected at Stage III they have a worse prognosis due to intrinsic chemoresistance (98, 99). Similarly, Ki67 staining has found that while indeed LGSC and HGSC are markedly different, CCOC, ENOC, and especially MOC can have high proliferation rates (100).

Molecular genetics and cell of origin studies have illustrated that while type II tumors can perhaps be considered together, type I tumors are too heterogeneous and instead should be considered as different diseases. The only context where the dualistic model has relevance is for serous carcinoma of the ovary since the type II HGSC and type I LGSC have distinct carcinogenesis, different precursors, well defined molecular profiles and different morphology and prognosis that fit within the description of the model. In contrast, the cancers with an origin in endometriosis should be considered a distinct group, with a shared origin but not necessarily an indolent course or with type I genetic profiles. The very clear differences between MOC and other type I tumors, the uncertainty about the origin of MOC and the heterogeneity of its mutational spectrum, suggests a separate classification for this entity. The wide variety in clinical behavior for MOC also suggests it should never be considered as an indolent “type I” tumor, although within MOC there may be indolent subgroups. Therefore, future studies for the diagnosis and treatment of EOC should consider the subtypes as different diseases, and novel therapies should also stratify EOC on the molecular characteristics of each tumor. Molecular profiling may suggest further stratification within histological subtypes, such as the already clinically relevant separation of HGSC and LGSC.

## Author Contributions

All authors listed have made a substantial, direct and intellectual contribution to the work, and approved it for publication.

### Conflict of Interest Statement

The authors declare that the research was conducted in the absence of any commercial or financial relationships that could be construed as a potential conflict of interest.
